# Faunistic and bibliographical inventory of moth flies from Ukraine (Diptera, Psychodidae)

**DOI:** 10.3897/zookeys.693.13652

**Published:** 2017-08-23

**Authors:** Jan Ježek, Pavel Chvojka, Peter Manko, Jozef Oboňa

**Affiliations:** 1 Department of Entomology, Cirkusová 1740, CZ – 193 00 Praha 9; 2 Horní Počernice, Czech Republic; 3 Department of Ecology, Faculty of Humanities and Natural Sciences, University of Prešov, 17. novembra 1, SK – 081 16 Prešov, Slovakia

**Keywords:** Diptera, faunistics, first records, Psychodidae, Phlebotominae, Sycoracinae, Psychodinae, Ukraine

## Abstract

All important published records for 11 moth fly species known so far from Ukraine are reviewed (Phlebotominae 10 species, Psychodinae 1 species). Occurrences of two problematic taxa, Phlebotomus (Adlerius) brevis Theodor & Mesghali, 1964 and P. (Larroussius) major major Annandale, 1910, and some synonymies are discussed. *Threticusnegrobovi* Vaillant, 1972 must be deleted for Ukraine (misstatement). First records of 34 species of Psychodinae (tribes Mormiini, Paramormiini, Psychodini, Pericomaini) and one of Sycoracinae from Ukraine are also listed.

## Introduction

Moth flies (Psychodidae) are represented only by 11 species previously recorded in Ukraine (mainly Perfil´ev 1966; Lewis 1982; Artemiev and Neronov 1984; Vaillant 1972; Wagner 1990, 2013). Most of these species are members of subfamily Phlebotominae: Phlebotomus (Phlebotomus) papatasi (Scopoli, 1786), P. (Adlerius) balcanicus Theodor, 1958, P. (A.) brevis Theodor & Mesghali, 1964 (doubtful evidence for Ukraine), P. (A.) longiductus Parrot, 1928, P. (Larroussius) neglectus Tonnoir, 1921, P. (L.) major major Annandale, 1910 (doubtful evidence for Ukraine), P. (L.) perfiliewi perfiliewi Parrot 1930, P. (Paraphlebotomus) alexandri Sinton, 1928, P. (P.) similis Perfil´ev, 1963 and *Sergentomyiaminutaminuta* (Rondani, 1843); only one species from subfamily Psychodinae has been listed: *Threticusnegrobovi* Vaillant, 1972. This is probably due to erroneous distribution data. In the current paper, the fauna of moth flies in Ukraine is enriched with 35 newly recorded species. The number of known species of Psychodidae from Ukraine is elevated to 43 .

## Materials and methods

The material presented here comes from two different sampling campaigns (see Table 1). The first part of the material was taken during field work as an implementation of the project “Ephemeroptera, Plecoptera, Diptera biodiversity trip along the Uzh river, Ukraine“ (2015 and 2016). The material was collected in the upper part of the Uzh River basin, in the eastern Ukraine (Transcarpathia) and preserved in 75% ethanol. All samples were collected by sweep netting from vegetation along streams (Uzh River and its tributaries) and lakes by Jozef Oboňa, Peter Manko and Ľuboš Hrivniak. Captured moth flies were mounted on slides in Canada balsam in laboratory – 51 slides. The same collecting method was used by Pavel Chvojka during sampling trip to eastern Carpathians (1996) – 19 slides. All material, determined by the first author, is deposited in the National Museum, Natural History Museum, Department of Entomology, Prague, Czech Republic (NMPC). Slides are numbered by Inv. No. = Inventory Slide Number of the family Psychodidae (Tkoč et al. 2014). Nomenclature utilised is according to Perfil´ev (1966), Lewis (1982) and Artemiev and Neronov (1984) for Phlebotominae; for Psychodinae and Sycoracinae the nomenclature is modified from Vaillant (1972) and Wagner (1990, 2013) using the classifications of Oboňa and Ježek (2014) and Kroča and Ježek (2015).

**Table 1.** List of collecting sites.

Site name	Latitude/N, Longitude/E
Hropynets' Stream	48°11'54.0", 24°16'42.0"
Kamenychky Stream, below Novoselytsya	48°48'36.9", 22°28'33.4"
Kevele Stream, left tributary of Chorna Tysa River	48°11'11.0", 24°18'37.0"
Lubnya River, below Lubnya	49°00'36.9", 22°43'27.8"
Lyuta River, above Chornoholova	48°51'43.1", 22°36'21.4"
Paporotnyi Stream, in Stuzhytsya	49°01'40.0", 22°35'17.1"
Preluchnyi Stream, bridge above the water reservoir	48°46'22.0", 22°52'32.6"
Pylypets' River	48°40'12.0", 23°20'17.0"
Shypot River, below hydroelectric power plant	48°42'58.2", 22°49'20.4"
Shypotyk Stream, tributary of the Shypot River near hydroelectric power plant	48°44'23.7", 22°50'18.4"
Strychavka Stream, below Strychava	48°56'43.6", 22°28'17.0"
Sukchyi Stream, above Kam'yanytsya	48°42'46.2", 22°25'24.9"
Synevir–Polyans'ke Lake	48°37'00.0", 23°40'54.0"
Tereblya River, below Kolochava	48°25'22.0", 23°41'36.0"
Tikhyi Stream, above Stuzhytsya	49°02'09.2", 22°34'43.5"
tributary of the Uzh River, near Zahorb	49°00'41.9", 22°38'39.1"
Turiya River above Simer	48°43'41.5", 22°32'41.9"
Uzh River, at the last building in Stuzhytsya	48°40'49.9", 22°24'09.6"
Uzh River, Nevyts’ke near bridge	48°40'49.9", 22°24'09.6"
Uzh River, above Uzhgorod	48°36'37.5", 22°13'49.6"

**The following abbreviations are used in the paper**:

**C** P. Chvojka leg.,

**H** Ľ. Hrivniak leg.,

**J** J. Ježek det.,

**Ma** P. Manko leg.,

**O** J. Oboňa leg.,

**NMPC** collections of the National Museum Prague, Czech Rep.,

**M** male,

**F** female.

## Results

### Faunistic records

#### Psychodidae

##### Phlebotominae

###### *Phlebotomus* Rondani, 1840 (sensu Sabrosky, 1999)

####### *Phlebotomus* s. str.

######## 1. Phlebotomus (Phlebotomus) papatasi

Taxon classificationAnimaliaDipteraPsychodidae

(Scopoli, 1786)

F609690D-C186-5B57-AC28-1014AFFAC4BF

######### Published records.

Perfil´ev (1966): 252, 254; Lewis (1982): 138, 140; Artemiev and Neronov (1984): 39, 40; Wagner (1990, 2013): 13.

######### Distribution.

Afghanistan, Albania, Algeria, Azerbaijan, Bulgaria, Crete, Crimea, Croatia, Cyprus, Egypt, Ethiopia, France (southern), Georgia, Greece, Hungary, India, Iran, Iraq, Israel, Italy, Jordan, Kazakhstan (southern), Kuwait, Libya, Malta, Moldavia, Montenegro, Morocco, Oman, Pakistan, Portugal, Romania, Sardinia, Saudi Arabia, Serbia, Spain, Sudan, Syria, Tunisia, Turkey, Ukraine (southern), Yemen (Perfil´ev 1966; Lewis 1982; Artemiev and Neronov 1984; Wagner 1990, 2013).

####### *Adlerius* Nitzulescu, 1931

######## 2. Phlebotomus (Adlerius) balcanicus

Taxon classificationAnimaliaDipteraPsychodidae

Theodor, 1958

8C1BAE4E-56BA-54F0-8B59-DCDC0E481F6F

######### Published records.

Perfil´ev (1966) as *Phlebotomuschinensisbalcanicus* Theodor, 1958: 316, 317; Lewis (1982): 165; Artemiev and Neronov (1984): 114; Wagner (1990, 2013): 17.

######### Distribution.

Azerbaijan, Bulgaria, Caucasus, Crimea, Greece, Iran (north-western), Romania, Turkey, Ukraine, former Yugoslavia (Perfil´ev 1966; Lewis 1982; Artemiev and Neronov 1984; Wagner 1990, 2013).

######## Phlebotomus (Adlerius) brevis

Taxon classificationAnimaliaDipteraPsychodidae

Theodor & Mesghali, 1964

9C4E73A3-868A-52E6-9EB9-F3FC1387BAAE

(doubtful evidence for Ukraine, see quite different views below)
Syn. Phlebotomuschinensisismailicus Perfil´ev, 1966

######### Published records.

Perfil´ev (1966) as *Phlebotomuschinensisismailicus* Perfil´ev, 1966: 314; Lewis (1982): 165; Artemiev and Neronov (1984): 115; Wagner (1990, 2013): 17.

######### Comments on distribution.

Artemiev and Neronov (1984): only Iran, Southern Caucasus, Turkey; Perfil´ev´s statement Izmail is erroneous (published as *ismailicus*). See the full synonymy in Lewis (1982). Wagner (1990, 2013) lists records from the republic of Moldova and Ukraine; however, these records are **not verified so far**.

######## 3 . Phlebotomus (Adlerius) longiductus

Taxon classificationAnimaliaDipteraPsychodidae

Parrot, 1928

E8EAD19F-2BBC-5EAC-A2F1-2E21A4BB0B6D

Syn. Phlebotomus (Adlerius) tauriae Perfil´ev, 1966: 312, 314, Crimea

######### Published records.

Perfil´ev (1966): 310, 312; Lewis (1982): 167; Artemiev and Neronov (1984): 121; Wagner (1990, 2013): 18.

######### Comments on distribution.

Romania Perfil´ev (1966): as *Phlebotomuschinensislongiductus* Parrot, 1928 – occurrence only in Central Asia. Lewis (1982) and Artemiev and Neronov (1984): Afghanistan (northern and central), Crimea, Kazakhstan, Romania, Ukraine (southern); Caucasus (northern) and Central Asia. Wagner (1990, 2013) lists Afghanistan, Kazakhstan, Moldova, Romania and the Ukraine.

####### *Larroussius* Nitzulescu, 1931

######## 4. Phlebotomus (Larroussius) neglectus

Taxon classificationAnimaliaDipteraPsychodidae

Tonnoir, 1921

9B6B2F60-7391-55B1-9512-697AF2D9B6BF

Syn. Phlebotomus (Larroussius) major krimensis Perfil´ev, 1966

######### Published records.

Perfil´ev (1966): 282, 285; Lewis (1982): 156, 157 (as *majorkrimensis*), 157 (as *majorneglectus*); Artemiev and Neronov (1984): 93, 95 (as *neglectus*); Wagner (1990, 2013): 16, (as *majorkrimensis*), 16 (as *majorneglectus*).

######### Comments on distribution.

Perfil´ev (1966): southern coast of Crimea; Lewis (1982): *krimensis* – Crimea, *neglectus* – Albania, Dalmatia, Italy; Artemiev and Neronov (1984): *neglectus* – Italy, Balkan Peninsula, Crimea, Turkey, Caucasus (southern), former Palestine, ?Iran (north-western). Wagner (1990, 2013) lists the species (as *majorkrimensis*) from Crimea, Ukraine, and as *majorneglectus* from Albania, Austria, Greece (Crete), Italy (Sicily), Romania, and former Yugoslavia.

######## Phlebotomus (Larroussius) major major

Taxon classificationAnimaliaDipteraPsychodidae

Annandale, 1910

4A9B0460-2CF5-5F06-8725-2B00566526AA

major (doubtful evidence for Ukraine, see quite different views below)

######### Published records.

Perfil´ev (1966): 280; Lewis (1982): 157; Artemiev and Neronov (1984): 92, 93; Wagner (1990, 2013): 16.

######### Comments on distribution.

Perfil´ev (1966): India only. Lewis (1982): India, Nepal, Pakistan. Artemiev and Neronov (1984): Afghanistan, India (southern slopes of the Himalayas), Nepal, Pakistan (northern). Wagner (1990) lists Afghanistan, India, Iran, and the Oriental Region. Wagner (2013) lists the species from Albania, Austria, Crete, Romania, Sicily, Ukraine, and former Yugoslavia; however, these records have **not been verified.**

######## 5. Phlebotomus (Larroussius) perfiliewi perfiliewi

Taxon classificationAnimaliaDipteraPsychodidae

Parrot, 1930

FD4AB0AE-CF2B-5228-BE55-D6486F1A7BDB

######### Published records.

Perfil´ev (1966): 289, 291; Lewis (1982): 160; Artemiev and Neronov (1984): 98; Wagner (1990, 2013): 16.

######### Distribution.

Albania, Algeria, Bosnia and Herzegovina, Crete, Crimea, Croatia, Cyprus, France (south-eastern), Georgia, Greece, Hungary, Italy, Libya, Macedonia, Malta, Moldova, Montenegro, Morocco, Portugal, Romania, Sardinia, Serbia, Sicily, Spain, Tunisia, Turkey, and Ukraine (Perfil´ev 1966; Lewis 1982; Artemiev and Neronov 1984; Wagner 1990, 2013).

####### *Paraphlebotomus* Theodor, 1948

######## 6. Phlebotomus (Paraphlebotomus) alexandri

Taxon classificationAnimaliaDipteraPsychodidae

Sinton, 1928

A46B670C-8855-5142-B999-04A67B16C7D4

######### Published records.

Perfil´ev (1966): 264, 267; Lewis (1982): 143; Artemiev and Neronov (1984): 45; Wagner (1990, 2013): 14.

######### Distribution.

Afghanistan, Algeria, Armenia, Azerbaijan, China (western), Crimea, Cyprus, Djibouti, Ethiopia, Georgia, Greece, India, Iran, Iraq, Israel, Kazakhstan (southern), Moldova, Mongolia, Morocco, Pakistan, Romania, Saudi Arabia, Spain, Sudan, Tunisia, Turkey, Ukraine, United Arab Emirates, Yemen; northern Sahara, Caucasus (southern), Near and Middle East, Central and Eastern Asia; Afrotropical and Oriental Regions (Perfil´ev 1966; Lewis 1982; Artemiev and Neronov 1984; Wagner 1990, 2013).

######## 7. Phlebotomus (Paraphlebotomus) similis

Taxon classificationAnimaliaDipteraPsychodidae

Perfil´ev, 1963

7D4B5371-DCAB-571B-A4C4-F1819CB75718

Sensu Artemiev and Neronov (1984)
Syn: Phlebotomus (Paraphlebotomus) sergenti similis Perfil´ev, 1963

######### Published records.

Perfil´ev (1966): 276; Lewis (1982): 148 – as *sergentisimilis*; Artemiev and Neronov (1984): 58, 59 – as *similis*.

######### Comments on distribution.

Perfil´ev (1966): southern coast of Crimea and southern Ukraine, Russia, northern Caucasus. Lewis (1982): same, Azerbaijan, Uzbekistan. Artemiev and Neronov (1984): Crimea, southern Ukraine, ?northern Caucasus, Albania, Romania, and former Yugoslavia.

####### *Sergentomyia* França & Parrot, 1920

######## 8. Sergentomyia minuta minuta

Taxon classificationAnimaliaDipteraPsychodidae

(Rondani, 1843)

DA385593-7180-5C5F-A81E-7CEC46543EB9

######### Published records.

Perfil´ev (1966): 323, 327; Wagner (1990): 22.

######### Distribution.

Crimea, France, Greece, Italy, Malta, Sardinia, Sicily, Spain, former Yugoslavia (Perfil´ev 1966; Wagner 1990).

##### PSYCHODINAE

###### MORMIINI

####### MORMIINA

######## *Oomormia* Ježek, 1984

######### 9. Oomormia andrenipes

Taxon classificationAnimaliaDipteraPsychodidae

(Strobl, 1910)

B4754F30-29A9-5782-8221-6A6BB5030B99

########## Material examined.

Rachiv, tributary of Chorna Tysa (Kvasy), 7.vi.1996, 1M, C leg., slide Inv. No. 12216, NMPC.

########## Distribution.

A quite rare species known from Austria, Bosnia, Czech Republic, Great Britain, Slovakia, and Slovenia (Ježek and Omelková 2012; Wagner 2013). **New species for Ukraine**.

###### PARAMORMIINI

####### PARAMORMIINA

######## *Jungiella* Vaillant, 1972

######### *Jungiella* s. str.

########## 10. Jungiella (Jungiella) hygrophila

Taxon classificationAnimaliaDipteraPsychodidae

Ježek, 1987

1386BAE2-6423-579E-95D0-0A91D003A7B5

########### Material examined.

Lyuta River, above Chornoholova, 27.v.2016, 1M, O Ma H leg., slide Inv. No. 22612, NMPC.

########### Distribution.

Probably a central European species, uncommon, known only from the Czech Republic, Poland, and Slovakia (Ježek 1987; Ježek and Omelková 2012; Wagner 2013; Oboňa and Ježek 2014). **New species for Ukraine**.

########## 11. Jungiella (Jungiella) valachica

Taxon classificationAnimaliaDipteraPsychodidae

(Vaillant, 1963)

2B5F6B81-07DC-5F2B-B68B-D7964A48E0F2

########### Material examined.

Lyuta River, above Chornoholova, 27.v.2016, 1M, O Ma H leg., slide Inv. No. 22610, NMPC.

########### Distribution.

A quite sporadic species, known from Austria, Bosnia and Herzegovina, Croatia, Czech Republic, Poland, Serbia, Slovakia, and Switzerland (Krek 1999; Ježek and Omelková 2012; Kvifte et al. 2013; Wagner 2013; Oboňa and Ježek 2014). **New species for Ukraine.**

######### *Psychogella* Ježek, 1983

########## 12. Jungiella (Psychogella) bohemica

Taxon classificationAnimaliaDipteraPsychodidae

Ježek, 1979

D968E20B-5DDD-5FFF-B586-ED8447B2B774

########### Material examined.

Uzh River, Nevyts’ke near bridge, 10.viii.2015, 1M, O Ma H leg., slide Inv. No. 22502, NMPC.

########### Distribution.

A rare species, know only from Czech Republic, Greece, and Germany (Ježek 1979; Ježek and Goutner 1995; Wagner 2013). **New species for Ukraine.**

######### *Parajungiella* Vaillant, 1972

########## 13. Parajungiella longicornis

Taxon classificationAnimaliaDipteraPsychodidae

(Tonnoir, 1919)

17CE2BD2-3DB7-57FE-876E-2952A6BE6126

########### Material examined.

Lyuta River, above Chornoholova, 27.v.2016, 1M, O Ma H leg., slide Inv. No. 22611, NMPC.

########### Distribution.

Generally, a very common European and west Siberian species (Ježek 1992). In Europe, known from Austria, Belgium, Bosnia and Herzegovina, Croatia, Czech Republic, Denmark, Finland, France, Germany, Great Britain, Greece, Hungary, Ireland, Norway, Poland, Russia, Slovakia, Sweden, and the Netherlands (Ježek 1992; Ježek and Goutner 1995; Krek 1999; Kvifte et al. 2011, 2013). **New species for Ukraine.**

####### TRICHOPSYCHODINA

######## *Philosepedon* Eaton, 1904

######### *Philothreticus* Krek, 1999

########## 14. Philosepedon (Philothreticus) soljani

Taxon classificationAnimaliaDipteraPsychodidae

Krek, 1971

B6ADFCCA-079D-5066-9A71-75305BE7DD9B

########### Material examined.

Hropynets’ Stream, 7.vi.1996, 1M, C leg., slide Inv. No. 12217, NMPC.

########### Distribution.

A very rare species, so far known only from Bosnia and Herzegovina, Czech Republic, Finland, and Slovenia (Krek 1999; Salmela et al. 2014; Kroča and Ježek 2015). **New species for Ukraine.**

######### *Threticus* Eaton, 1904

########## Threticus negrobovi

Taxon classificationAnimaliaDipteraPsychodidae

Vaillant, 1972

7EEA4126-CD03-52D1-AFF6-612D1DF830DF

########### Published records.

Vaillant (1972): 104 (Taberda in Russian Caucasus, not Ukraine, compare with Wagner 1990, 2013)

########### Distribution.

Very rare mountain species, so far known from Czech Republic, Slovenia, Slovakia, Russia, Ukraine (**error**), Abkhazia and Georgia (Ježek 2004; Kroča and Ježek 2015).

########### Note.

*Threticusnegrobovi* Vaillant, 1972 was mentioned in the Palaearctic catalogue of moth flies from South European territory (Ukraine) based on a record from Teberda village (Wagner 1990; Taberda 2000 m a.s.l. sensu Vaillant 1972). This village is, however, in the Russian part of the Caucasus and the listing from the Ukraine must be regarded as an error.

###### PSYCHODINI

####### *Chodopsycha* Ježek, 1984

######## 15. Chodopsycha lobata

Taxon classificationAnimaliaDipteraPsychodidae

(Tonnoir, 1940)

157820A7-D48C-5783-807E-C744B1E1DB32

######### Material examined.

Preluchnyi Stream, bridge above the water reservoir, 10.viii.2015, 1F, O Ma H leg., slide Inv. No. 22513, NMPC.

######### Distribution.

A very common European (Transcaucasian) species, present in lowlands as well as in hills and mountains; associated with fungi. In Europe, known from Belgium, Bulgaria, France, Germany, Great Britain, Hungary, Ireland, Italy, Norway, and Slovenia (Kvifte et al. 2011; Ježek and Omelková 2012; Ježek et al. 2013). **New species for Ukraine.**

####### *Logima* Eaton, 1904

######## 16. Logima satchelli

Taxon classificationAnimaliaDipteraPsychodidae

(Quate, 1955)

4196F0FB-043C-51C8-8B27-8C2615F5D0B9

######### Material examined.

Preluchnyi Stream, bridge above the water reservoir, 10.viii.2015, 1F, O Ma H leg., slide Inv. No. 22515; Shypotyk Stream, tributary of the Shypot River near hydroelectric power plant, 11.viii.2015, 1F, O Ma H leg., slide Inv. No. 22500, all NMPC.

######### Distribution.

A common Holarctic species. In Europe, known from e.g. Austria, Czech Republic, Ireland, Italy, Norway, Russia, Slovakia, Slovenia, Sweden, the Netherlands, and former Yugoslavia; Canada, U.S.A. (Ježek and Goutner 1995; Svensson 2009; Kvifte et al. 2011). **New species for Ukraine.**

####### *Psycha* Ježek, 1984

######## 17. Psycha grisescens

Taxon classificationAnimaliaDipteraPsychodidae

(Tonnoir, 1922)

04A033D8-A9F9-5611-821A-35C9ACAA44FE

######### Material examined.

Synevir – Polyans’ke Lake, 3.vi.1996, 1F, C leg., slide Inv. No. 122241; Hropynets’ Stream, 7.vi.1996, 1F, C leg., slide Inv. No. 12221, all NMPC.

######### Distribution.

A European and North African species. Recorded from Algeria, Austria, Belgium, Bosnia and Herzegovina, Czech Republic, Denmark, the Faroe Islands, France, Finland, Germany, Great Britain, Greece, Hungary, Ireland, Italy, Mallorca, Morocco, the Netherlands, Norway, Slovakia, Slovenia, Sweden, Tunisia, and Turkey (Andersen 1999; Ježek 2004; Ježek and Yağci 2005; Salmela et al. 2014; Kvifte et al. 2016). **New species for Ukraine.**

####### *Psychodocha* Ježek, 1984

######## 18. Psychodocha cinerea

Taxon classificationAnimaliaDipteraPsychodidae

(Banks, 1894)

58F739B7-F376-571F-8C9A-C04BBFBA98E3

######### Material examined.

Preluchnyi Stream, bridge above the water reservoir, 10.viii.2015, 1F, O Ma H leg., slide Inv. No. 225145, NMPC.

######### Distribution.

A very common cosmopolitan species. Known from Austria, Azores, Belgium, Bosnia and Herzegovina, Bulgaria, Canary Islands, Cyprus, Czech Republic, Denmark, Finland, France, Germany, Great Britain, Greece, Hungary, Ireland, Italy (incl. Sardinia), Madeira, the Netherlands, Norway, Poland, Romania, Russia, Serbia, Slovakia, Slovenia, Spain, Sweden, Switzerland, and Turkey; Abkhazia, Afghanistan, Africa mer., Algeria, Argentina, Australia, Azores, Brazil, Canada, Chile, Iran, Israel, Juan Fernandéz Islands, New Zealand, Puerto Rico Islands, Tunisia, U.S.A. (Krek 1985; Ježek and Goutner 1995; Ježek and Yağci 2005; Kvifte et al. 2011; Wagner 1990, 2013; Salmela et al. 2014). **New species for Ukraine.**

######## 19. Psychodocha gemina

Taxon classificationAnimaliaDipteraPsychodidae

(Eaton, 1904)

FE32E812-9428-5859-A8A7-4131045677ED

######### Material examined.

Preluchnyi Stream, bridge above the water reservoir, 10.viii.2015, 1M, O Ma H leg., slide Inv. No. 22512; Shypot River, below the hydroelectric power plant, 10.viii.2015, 1F, O Ma H leg., slide Inv. No. 22517; Lyuta River, above Chornoholova, 11.viii.2015, 1F, O Ma H leg., slide Inv. No. 22492; Uzh River, at the last building in Stuzhytsya, 12.viii.2015, 1F, O Ma H leg., slide Inv. No. 22507, all NMPC.

######### Distribution.

A common European species. Recorded from Austria, Belgium, Bosnia and Herzegovina, Croatia, Czech Republic, Great Britain, Denmark, France, Finland, Germany, Greece, Hungary, Ireland, Norway, Romania, Serbia, Slovakia, Slovenia, Spain, Switzerland, and the Netherlands (Ježek and Goutner 1995; Krek 1999; Ježek 2002; Kvifte et al. 2011, 2013; Salmela et al. 2014). **New species for Ukraine.**

####### *Psychodula* Ježek, 1984

######## 20. Psychodula minuta

Taxon classificationAnimaliaDipteraPsychodidae

(Banks, 1894)

6A54CBC9-E611-5074-AD1A-1712D984DA84

######### Material examined.

Uzh River, at the last building in Stuzhytsya, 12.viii.2015, 1M, O Ma H leg., slide Inv. No. 22510, NMPC.

######### Distribution.

A generally common Holarctic species. Recorded from Abkhazia, Austria, Belgium, Britain, Bulgaria, Cyprus, Czech Republic, Denmark, France, Finland, Germany, Greece, Hungary, Ireland, Italy (incl. Sardinia), Madeira, the Netherlands, Norway, Romania, Slovakia, Slovenia, Spain (incl. Balearic Islands), Sweden, and Switzerland; Canada, Syria, U.S.A. (Ježek 1990; Wagner 2013; Salmela et al. 2014). **New species for Ukraine.**

####### *Psychomora* Ježek, 1984

######## 21. Psychomora mycophila

Taxon classificationAnimaliaDipteraPsychodidae

(Vaillant, 1988)

DB18866A-F457-53A6-AF2D-C06BAA1971DF

(syn. *vanharai* Ježek, 1995)

######### Material examined.

Shypotyk Stream, tributary of the Shypot River near hydroelectric power plant, 11.viii.2015, 1M, O Ma H leg., slide Inv. No. 22497, NMPC.

######### Distribution.

A rare species associated with fungi, so far known only from Czech Republic, France, Slovakia, Slovenia, and Switzerland (Ježek and Omelková 2012). **New species for Ukraine.**

######## 22. Psychomora trinodulosa

Taxon classificationAnimaliaDipteraPsychodidae

(Tonnoir, 1922)

0CB59D43-5062-54FB-8C3A-ACD44D392FA6

######### Material examined.

Shypotyk Stream, tributary of Shypot River near hydroelectric power plant, 11.viii.2015, 1F, O Ma H leg., slide Inv. No. 22499, NMPC.

######### Distribution.

A very common Holarctic species. Known from Austria, Belgium, Bulgaria, Croatia, Czech Republic, Denmark, Finland, France, Germany, Great Britain, Greek mainland, Hungary, Ireland, Italy, the Netherlands, Norway, Poland, Romania, Russia, Sardinia, Slovakia, Slovenia, Spanish mainland, and Sweden; Algeria, U.S.A. (Ježek 1990; Wagner 2013; Salmela et al. 2014). **New species for Ukraine.**

####### *Tinearia* Schellenberg, 1803

######## 23. Tinearia alternata

Taxon classificationAnimaliaDipteraPsychodidae

(Say, 1824)

A19042B8-A769-5A9C-9CB5-C3F4C39F40FA

######### Material examined.

Uzh River, Nevyts’ke near bridge, 10.viii.2015, 1F, O Ma H leg., slide Inv. No. 22501, NMPC.

######### Distribution.

A cosmopolitan species, generally very common. Known from Austria, Balearic Is., Belgium, Bulgaria, Canary Is., Crete, Croatia, Cyprus, Czech Republic, Denmark, Finland, France, Germany, Great Britain, Greek mainland, Hungary, Ireland, Italy, Madeira, Norway, Poland, Romania, Sardinia, Slovenia, Spanish mainland, Sweden, Switzerland, the Netherlands, and Norway; Afghanistan, whole Africa, Australia, Bangladesh, Borneo, Canal Zone, Cape Verde Islands, Philippines, Hawaii, India, Jamaica, Japan, Macquarie Islands, Malaysia, Micronesia, Mongolia, New Zealand, North and South America, North Korea, Puerto Rico, Ryukyu Islands, Samoa, Socotra Island, Taiwan, Trinidad (Ježek 1981; Ježek and van Harten 1996; Kvifte et al. 2011; Ježek and Tkoč 2012; Wagner 2013). **New species for Ukraine.**

###### PERICOMAINI

####### *Berdeniella* Vaillant, 1976

######## 24. Berdeniella kocii

Taxon classificationAnimaliaDipteraPsychodidae

Ježek, 2006

2A31FE5E-CAB7-5E83-B9E2-D6FD4D23113C

######### Material examined.

Shypot River, below hydroelectric power plant, 10.viii.2015, 1M, O Ma H leg., slide Inv. No. 22519; Shypotyk Stream, tributary of the Shypot River near hydroelectric power plant, 11.viii.2015, 1M, O Ma H leg., slide Inv. No. 22498; Tikhyi Stream, above Stuzhytsya, 12.viii.2015, 1M, O Ma H leg., slide Inv. No. 22523; tributary of the Uzh River, near Zahorb, 12.viii.2015, 1M, O Ma H leg., slide Inv. No. 22524; Paporotnyi Stream, in Stuzhytsya, 12.vii.2015, 1M, O Ma H leg., slide Inv. No. 22495 – all slides deposited in NMPC.

######### Distribution.

A rare mountain species, known only from Czech Republic and Slovakia (Ježek 2006, 2009; Ježek and Omelková 2012). **New species for Ukraine.**

######## 25. Berdeniella matthesi

Taxon classificationAnimaliaDipteraPsychodidae

(Jung, 1954)

BE21F5D1-B741-5322-88D6-984E44035461

######### Material examined.

Synevir – Polyans’ke Lake, 3.vi.1996, 1M, C leg., slide Inv. No. 12223, NMPC.

######### Distribution.

A locally common species in Europe, the knowledge on its distribution is, however, quite poor: so far, it has been collected in Austria, Czech Republic, Germany, Italy, and Slovakia (Ježek and Omelková 2012; Kroča and Ježek 2015). **New species for Ukraine.**

######## 26. Berdeniella stavniensis

Taxon classificationAnimaliaDipteraPsychodidae

(Krek, 1969)

D09BFAF2-A14C-51E2-B765-B9860C127266

######### Material examined.

Synevir – Polyans’ke Lake, 3.vi.1996, 1M, C leg., slide Inv. No. 12222; Hropynets’ Stream, 7.vi.1996, 1M, C leg., slide Inv. No. 12219, all NMPC.

######### Distribution.

European species, apparently not quite common, known from Austria, Bosnia and Herzegovina, Czech Republic, France, Germany, Serbia, and Slovakia (Krek 1999; Ježek 2003). **New species for Ukraine.**

######## 27. Berdeniella vimmeri

Taxon classificationAnimaliaDipteraPsychodidae

Ježek, 1997

940384CA-BD90-5CA6-A552-170F8B7AC439

######### Material examined.

Hropynets’ Stream, 7.vi.1996, 1M, C leg., slide Inv. No. 12233, NMPC.

######### Distribution.

A central European species (knowledge of distribution is poor so far); known from Czech Republic and Slovakia (Ježek 1997, 2009; Ježek and Omelková 2012). **New species for Ukraine.**

####### *Clytocerus* Eaton, 1904

######## *Boreoclytocerus* Duckhouse, 1978

######### 28. Clytocerus (Boreoclytocerus) longicorniculatus

Taxon classificationAnimaliaDipteraPsychodidae

Krek, 1987

3B222FCF-C2A4-5340-8076-66C3FE232453

########## Material examined.

Sukchyi Stream, above Kam’yanytsya, 12.viii.2015, 1M, O Ma H leg., slide Inv. No. 22521, NMPC.

########## Distribution.

Probably not common European species. Known only from Bosnia and Herzegovina, Czech Republic, and Slovakia (Krek 1999; Ježek 2009; Ježek et al. 2013). **New species for Ukraine.**

######### 29. Clytocerus (Boreoclytocerus) ocellaris

Taxon classificationAnimaliaDipteraPsychodidae

(Meigen, 1804)

7D55E9D6-A890-5372-973E-FE3D476CD3B1

########## Material examined.

Lyuta River, above Chornoholova, 11.viii.2015, 1M, O Ma H leg., slide Inv. No. 22494, NMPC.

########## Distribution.

A very common central and western European species. Known from Austria, Belgium, Great Britain, Bulgaria, Czech Republic, Denmark, Finland, France, Germany, Hungary, Ireland, Lithuania, the Netherlands, Norway, Poland, Sardinia, Slovenia, and Switzerland (Wagner 2013; Ježek et al. 2014). **New species for Ukraine.**

####### *Pericoma* Walker, 1856

######## *Pachypericoma* Vaillant, 1978

######### 30. Pericoma (Pachypericoma) blandula

Taxon classificationAnimaliaDipteraPsychodidae

Eaton, 1893

A47157F7-71DA-57CD-8CDC-14D275FCE4A8

########## Material examined.

Tereblya River, below Kolochava, 3.vi.1996, 1M, C leg., slide Inv. No. 12230; Hropynets’ Stream, 7.vi.1996, C leg., slide Inv. No. 12234; Uzh river, above Uzhgorod, 10.viii.2015, 1M, O Ma H leg., slide Inv. No. 22520; Tikhyi Stream, above Stuzhytsya, 12.viii.2015, 1M, O Ma H leg., slide Inv. No. 22484; tributary of the Uzh River, near Zahorb, 12.viii.2015, 1M, O Ma H leg., slide Inv. No. 22525; Paporotnyi Stream, in Stuzhytsya, 12.viii.2015, 1M, O Ma H leg., slide Inv. No. 22496; Sukchyi Stream, above Kam’yanytsya, 12.viii.201 5, 1M, O Ma H leg., slide Inv. No. 22522, all slides deposited in NMPC.

########## Distribution.

Species widespread in Europe and recorded also in Transcaucasia, Tunisia, and Morocco. In Europe, known from Austria, Belgium, Bosnia and Herzegovina, Croatia, Great Britain, Bulgaria, Czech Republic, Denmark, European Turkey, Finland, France, Germany, Greek mainland, Hungary, Ireland, Italy, Macedonia, Montenegro, the Netherlands, Norway, Poland, Romania, Russia, Sardinia, Serbia, Slovakia, Slovenia, Spanish mainland, Sweden, and Switzerland (Ježek 2004; Kvifte et al. 2011, 2013). **New species for Ukraine.**

######### 31. Pericoma (Pachypericoma) fallax

Taxon classificationAnimaliaDipteraPsychodidae

Eaton, 1893

9D5BCB8C-F918-57F1-8C1F-7DD3BB8E711C

########## Material examined.

Tereblya River, below Kolochava, 3.vi.1996, 1M, C leg., slide Inv. No. 12229; Shypot River, below hydroelectric power plant, 10.viii.2015, 1M, O Ma H leg., slide Inv. No. 22518; Kamenychky Stream, below Novoselytsya, 11.viii.2015, 1M, O Ma H leg., slide Inv. No. 22485; Tikhyi Stream, above Stuzhytsya, 12.viii.2015, 1M, O Ma H leg., slide Inv. No. 22483, all slides deposited in NMPC.

########## Distribution.

A European and western Siberian species. In Europe, known from Belgium, Bosnia and Herzegovina, Czech Republic, France, Germany, Great Britain, Greece, Hungary, Ireland, Italy, Macedonia, Montenegro, the Netherlands, Slovenia, and Spain (Ježek 1992, 2004; Wagner 2013). **New species for Ukraine.**

######### 32. Pericoma (Pachypericoma) nielseni

Taxon classificationAnimaliaDipteraPsychodidae

Kvifte, 2010

C0B727FF-EFBE-5F6D-B19E-563080B0ED88

Syn: **Pericomaformosa** Nielsen, 1964

########## Material examined.

Tereblya River, below Kolochava, 3.vi.1996, 1M, C leg., slide Inv. No. 12232; Uzh River, Nevyts’ke near bridge, 10.viii.2015, 1M, O Ma H leg., slide Inv. No. 22504, all in NMPC.

**Distribution**: Not common European species, known from Czech Republic, Denmark, Finland, France, Norway, and Slovakia (Ježek 2009; Kvifte 2010; Kvifte et al. 2011). **New species for Ukraine.**

######## *Pericoma* *s. str.*

######### 33. Pericoma (Pericoma) exquisita

Taxon classificationAnimaliaDipteraPsychodidae

Eaton, 1893

5BFDBC00-25A6-5D1B-B487-C6874FD37ED1

########## Material examined.

Uzh River, Nevyts’ke near bridge, 10.viii.2015, 1M, O Ma H leg., slide Inv. No. 22503; Lyuta River, above Chornoholova, 11.viii.2015, 1M, O Ma H leg., slide Inv. No. 22493; Uzh River, at the last building in Stuzhytsya, 12.viii.2015, 1M, O Ma H leg., slide Inv. No. 22509, all in NMPC.

########## Distribution.

Species widespread in Europe, North Africa, and Transcaucasia (Armenia). In Europe, known from Albania, Austria, Belgium, Bosnia and Herzegovina, Bulgaria, Czech Republic, Crete, Croatia, France, Germany, Great Britain, Greece, Hungary, Ireland, Italy, Macedonia, Montenegro, Poland, Serbia, Slovakia, Slovenia, and Spain (Ježek 2004, 2009; Kvifte et al. 2013; Wagner 2013; Oboňa and Ježek 2014). **New species for Ukraine**.

####### *Pneumia* Enderlein, 1935

######## 34. Pneumia crispi

Taxon classificationAnimaliaDipteraPsychodidae

(Freeman, 1953)

075BE766-24FD-57D5-B4CD-3A4B40F18A8B

######### Material examined.

Lubnya River, below Lubnya, 11.viii.2015, 1M, O Ma H leg., slide Inv. No. 22505, NMPC.

######### Distribution.

A rare European species. So far recorded from Bosnia and Herzegovina, Czech Republic, France, Germany, Great Britain, Greece, Hungary, Macedonia, Romania, and Slovakia (Ježek and Omelková 2012). **New species for Ukraine**.

######## 35. Pneumia gracilis gracilis

Taxon classificationAnimaliaDipteraPsychodidae

(Eaton, 1893)

06A79925-C2F2-54E8-8578-97A5AD614D9E

######### Material examined.

Strychavka Stream, below Strychava, 11.viii.2015, 1M, O Ma H leg., slide Inv. No. 22516; Tikhyi Stream, above Stuzhytsya, 12.viii.2015, 1M, O Ma H leg., slide Inv. No. 22482, all in NMPC.

######### Distribution.

A species published from several countries, not too frequent in Europe: Albania, Belgium, Bosnia and Herzegovina, Czech Republic, France, Germany, Great Britain, Greece, Italy, Serbia, Slovakia, and Slovenia; Transcaucasia: Abkhazia (Ježek 2002, 2004; Ježek et al. 2012). **New species for Ukraine**.

######## 36. Pneumia mutua

Taxon classificationAnimaliaDipteraPsychodidae

(Eaton, 1893)

1A6224B8-7D87-5AF7-85DA-848B940CADEB

######### Material examined.

Synevir – Polyans’ke Lake, 3.vi.1996, 1M, C leg., slide Inv. No. 12227; Hropynets’ Stream, 7.vi.1996, 1M, C leg., slide Inv. No. 12235, all in NMPC.

######### Distribution.

A common species distributed largely in western and central Europe, and Scandinavia; known from Austria, Belgium, Croatia, Czech Republic, Denmark, Finland, France, Germany, Great Britain, Hungary, Ireland, Italy, the Netherlands, Norway, Poland, Slovakia, and Slovenia (Kvifte et al. 2011, 2013; Oboňa and Ježek 2014). **New species for Ukraine.**

######## 37. Pneumia nubila

Taxon classificationAnimaliaDipteraPsychodidae

(Meigen, 1818)

9F216EBD-C566-52C2-B266-BB4163052009

######### Material examined.

Lyuta River, above Chornoholova, 11.viii.2015, 1M, O Ma H leg., slide Inv. No. 22489; 27.v.2016, 1M, O Ma H leg., slide Inv. No. 22609, all in NMPC.

######### Distribution.

A very common species, recorded from throughout Europe and the Canary Islands. Known from Austria, Belgium, Bosnia and Herzegovina, Bulgaria, Croatia, Czech Republic, Denmark, Finland, France, Germany, Great Britain, Greece, Hungary, Ireland, Italy, Luxembourg, Macedonia, Montenegro, Poland, Romania, Sardinia, Serbia, Slovakia, Slovenia, Spain, Switzerland, and the Netherlands (Ježek and Goutner 1995; Krek 1999; Ježek 2002; Kvifte et al. 2013). **New species for Ukraine.**

######## 38. Pneumia stammeri

Taxon classificationAnimaliaDipteraPsychodidae

(Jung, 1956)

34C27EEF-0401-5C98-90A2-F4C7BFB8093A

######### Material examined.

Pylypets’ River, 2.vi.1996, C leg., 1M, slide Inv. No. 12228; Synevir – Polyans’ke Lake, 3.vi.1996, C leg., 1M, slide Inv. No. 12226, all in NMPC.

######### Distribution.

A quite rare European species known from Austria, Belgium, Bosnia, Czech Republic, Denmark, France, Finland, Germany, Great Britain, Hungary, Norway, Slovakia, and Sweden (Ježek 2003; Kvifte et al. 2011; Salmela et al. 2014). **New species for Ukraine.**

######## 39. Pneumia trivialis

Taxon classificationAnimaliaDipteraPsychodidae

(Eaton, 1893)

DF0A586B-CB9B-5981-B842-EE235113A012

######### Material examined.

Lyuta River, above Chornoholova, 11.viii.2015, 1M, O Ma H leg., slide Inv. No. 22491; Tikhyi Stream, above Stuzhytsya, 12.viii.2015, 1M, O Ma H leg., slide Inv. No. 22481; tributary of the Uzh River, near Zahorb, 12.viii.2015, 1M, O Ma H leg., slide Inv. No. 22488, all slides deposited in NMPC.

######### Distribution.

A very common European species. Known from Austria, Belgium, Bosnia and Herzegovina, Croatia, Czech Republic, Denmark, Finland, France, Germany, Great Britain, Hungary, Ireland, the Netherlands, Norway, Poland, Serbia, Slovakia, Slovenia, Spain, Sweden, and Switzerland (Krek 1999; Ježek 2002; Kvifte et al. 2011, 2013). **New species for Ukraine.**

######## 40. Pneumia tjentistensis

Taxon classificationAnimaliaDipteraPsychodidae

(Krek, 1969)

B0D7E333-CF03-5403-99AF-60B5DF8E42B3

######### Material examined.

Hropynets’ Stream, 7.vi.1996, 1M, C leg., slide Inv. No. 12218, NMPC.

######### Distribution.

So far known only from Bosnia and Herzegovina, and Czech Republic (Krek 1999; Ježek and Omelková 2012). **New species for Ukraine.**

####### *Ulomyia* Walker, 1856

######## 41. Ulomyia cognata

Taxon classificationAnimaliaDipteraPsychodidae

(Eaton, 1893)

D2BA75A5-5854-5B57-ACB3-0C729A51A974

######### Material examined.

Synevir – Polyans’ke Lake, 3.vi.1996, 1M, C leg., slide Inv. No. 12225; Hropynets’ Stream, 7.vi.1996, 1M, C leg., slide Inv. No. 12220; Lubnya River, below Lubnya, 11.viii.2015, 1M, O Ma H leg., slide Inv. No. 22506; tributary of the Uzh River, near Zahorb, 12.viii.2015, 1M, O Ma H leg., slide Inv. No. 22487; Uzh River, at the last building in Stuzhytsya, 12.viii.2015, 1M, O Ma H leg., slide Inv. No. 22508, all slides deposited in NMPC.

######### Distribution.

A common European species known from Austria, Czech Republic, Finland, France, Germany, Great Britain, Italy, Lithuania, Poland, Slovakia, and Slovenia (Ježek and Omelková 2012; Salmela et al. 2014). **New species for Ukraine.**

######## 42. Ulomyia fuliginosa

Taxon classificationAnimaliaDipteraPsychodidae

(Meigen, 1804)

40C3DECE-3E46-5B83-83FB-D8808608EE8E

######### Material examined.

Preluchnyi Stream, bridge above the water reservoir, 10.viii.2015, 1M, O Ma H leg., slide Inv. No. 22511; Lyuta River, above Chornoholova, 11.viii.2015, 1M, O Ma H leg., slide Inv. No. 22490; Tikhyi Stream, above Stuzhytsya, 12.viii.2015, 1M, O Ma H leg., slide Inv. No. 22480; tributary of the Uzh River, near Zahorb; same, 12.viii.2015, 1M, O Ma H leg., slide Inv. No. 22486, all slides deposited in NMPC.

######### Distribution.

A very common and often frequently recorded European species, known from Austria, Belgium, Bosnia and Herzegovina, Croatia, Czech Republic, Denmark, Finland, France, Germany, Great Britain, Hungary, Ireland, Italy, Kosovo, Macedonia, the Netherlands, Norway, Poland, Romania, Slovakia, Slovenia, Spain, and Switzerland (Krek 1999; Ježek 2002; Kvifte et al. 2011, 2013; Salmela et al. 2014). **New species for Ukraine.**

##### SYCORACINAE

###### *Sycorax* Haliday in Curtis, 1839

####### 43. Sycorax silacea

Taxon classificationAnimaliaDipteraPsychodidae

Haliday in Curtis, 1839

1471BF65-1CBC-538B-9C3B-FD7890B56CFA

######## Material examined.

Turiya River above Simer, 27.v.2016, 1M, O Ma H leg., slide Inv. No. 22613, NMPC.

######## Distribution.

European species, locally common; known from Belgium, Bosnia and Herzegovina, Croatia, Czech Republic, Denmark, Finland, France, Germany, Great Britain, Hungary, Ireland, Italy, Norway, Poland, Romania, Slovakia Sweden, and Switzerland (Ježek 1996; Krek 1999; Kvifte et al. 2013; Wagner 2013; Salmela et al. 2014) . **New species for Ukraine.**

## Supplementary Material

XML Treatment for Phlebotomus (Phlebotomus) papatasi

XML Treatment for Phlebotomus (Adlerius) balcanicus

XML Treatment for Phlebotomus (Adlerius) brevis

XML Treatment for Phlebotomus (Adlerius) longiductus

XML Treatment for Phlebotomus (Larroussius) neglectus

XML Treatment for Phlebotomus (Larroussius) major major

XML Treatment for Phlebotomus (Larroussius) perfiliewi perfiliewi

XML Treatment for Phlebotomus (Paraphlebotomus) alexandri

XML Treatment for Phlebotomus (Paraphlebotomus) similis

XML Treatment for Sergentomyia minuta minuta

XML Treatment for Oomormia andrenipes

XML Treatment for Jungiella (Jungiella) hygrophila

XML Treatment for Jungiella (Jungiella) valachica

XML Treatment for Jungiella (Psychogella) bohemica

XML Treatment for Parajungiella longicornis

XML Treatment for Philosepedon (Philothreticus) soljani

XML Treatment for Threticus negrobovi

XML Treatment for Chodopsycha lobata

XML Treatment for Logima satchelli

XML Treatment for Psycha grisescens

XML Treatment for Psychodocha cinerea

XML Treatment for Psychodocha gemina

XML Treatment for Psychodula minuta

XML Treatment for Psychomora mycophila

XML Treatment for Psychomora trinodulosa

XML Treatment for Tinearia alternata

XML Treatment for Berdeniella kocii

XML Treatment for Berdeniella matthesi

XML Treatment for Berdeniella stavniensis

XML Treatment for Berdeniella vimmeri

XML Treatment for Clytocerus (Boreoclytocerus) longicorniculatus

XML Treatment for Clytocerus (Boreoclytocerus) ocellaris

XML Treatment for Pericoma (Pachypericoma) blandula

XML Treatment for Pericoma (Pachypericoma) fallax

XML Treatment for Pericoma (Pachypericoma) nielseni

XML Treatment for Pericoma (Pericoma) exquisita

XML Treatment for Pneumia crispi

XML Treatment for Pneumia gracilis gracilis

XML Treatment for Pneumia mutua

XML Treatment for Pneumia nubila

XML Treatment for Pneumia stammeri

XML Treatment for Pneumia trivialis

XML Treatment for Pneumia tjentistensis

XML Treatment for Ulomyia cognata

XML Treatment for Ulomyia fuliginosa

XML Treatment for Sycorax silacea
